# A Synchronous Motor Imagery Based Neural Physiological Paradigm for Brain Computer Interface Speller

**DOI:** 10.3389/fnhum.2017.00274

**Published:** 2017-05-29

**Authors:** Lei Cao, Bin Xia, Oladazimi Maysam, Jie Li, Hong Xie, Niels Birbaumer

**Affiliations:** ^1^Department of Computer Science, College of Information Engineering, Shanghai Maritime UniversityShanghai, China; ^2^Institute of Medical Psychology and Behavioral Neurobiology, University of TuebingenTuebingen, Germany; ^3^Werner Reichardt, Center for Integrative Neuroscience (System Neurophysiology), University of TuebingenTuebingen, Germany; ^4^Department of Computer Science and Technology, Tongji UniversityShanghai, China; ^5^IRCCS Fondazione Ospedale San CamilloVenezia, Italy

**Keywords:** brain computer interface (BCI), motor imagery (MI), Oct-o-spell paradigm, synchronous control, speller

## Abstract

Brain Computer Interface (BCI) speller is a typical BCI-based application to help paralyzed patients express their thoughts. This paper proposed a novel motor imagery based BCI speller with Oct-o-spell paradigm for word input. Furthermore, an intelligent input method was used for improving the performance of the BCI speller. For the English word spelling experiment, we compared synchronous control with previous asynchronous control under the same experimental condition. There were no significant differences between these two control methods in the classification accuracy, information transmission rate (ITR) or letters per minute (LPM). And the accuracy rates of over 70% validated the feasibility for these two control strategies. It was indicated that MI-based synchronous control protocol was feasible for BCI speller. And the efficiency of the predictive text entry (PTE) mode was superior to that of the Non-PTE mode.

## 1. Introduction

Brain-computer interface (BCI) system builds a communication bridge between the brain and the external world by transforming neural signals into control commands without body movement (Birbaumer et al., 1999; Pfurtscheller et al., 2000; Guger et al., 2003; Blankertz et al., 2004; Birbaumer, 2006; Wolpaw, 2014). For severely disabled patients, BCI can help them to manipulate external devices for communication (Sellers and Donchin, 2006; Nijboer et al., 2008; Mainsah et al., 2015).

Electroencephalogram (EEG) is commonly used for BCI. This technology is developed based on neurophysiological patterns such as event related potentials (ERPs) (Blankertz et al., 2011; Zhang et al., 2012; Jin et al., 2014; Yeom et al., 2014), slow cortical potentials (Mensh et al., 2004; Kübler and Birbaumer, 2008), event-related desynchronization/synchronization (Pfurtscheller and Neuper, 2006; Pfurtscheller et al., 2010; Lisi et al., 2014; Sandhya et al., 2014) and steady-state evoked potentials (SSVEPs) (Müller-Putz and Pfurtscheller, 2008; Wang et al., 2008; Allison et al., 2010). BCI speller is one of the common BCI-based applications that paralyzed patients use to express their thoughts (Allison and Pineda, 2003; Krusienski et al., 2008). In 1988, Farwell et al. proposed the P300-based BCI speller (Farwell and Donchin, 1988). P300 signal is detected using Oddball paradigm (Polich and Kok, 1995). The classical interface was composed of a 6 × 6 letter matrix including 26 characters, 9 numbers, and a SPACE symbol. Users can spell characters using P300-based target selection. In recent years, P300 spellers have been studied for improving signal processing (Kindermans et al., 2012; Throckmorton et al., 2013; Mainsah et al., 2014a; Speier et al., 2014, 2015) and paradigm designing (Combaz et al., 2012; Jin et al., 2012; Mainsah et al., 2014b; Shih et al., 2014; Li et al., 2015). Letter matrices were used to display optional targets, and different flash patterns were developed for improving P300-based classification accuracy and speed.

Cecotti proposed a SSVEP-based speller with five stimulus buttons including character groups (Cecotti, 2010). To spell a letter, user should focus on the corresponding stimuli. Volosyak et al. developed a SSVEP speller controlled using a virtual keyboard (Volosyak et al., 2011). Five commands were used to move the cursor and select the character in a virtual keyboard. Speed of the SSVEP-based speller is reduced due to the limitation of control commands. To solve this problem, 30 flicking stimulus with different frequencies were designated to the speller (Hwang et al., 2012). The average speed was 9.39 letter per minute (LPM), and the average rate of data transfer was 40.72 bits per minute. One disadvantage for these spellers is visual fatigue caused by prolonged gazing.

The Berlin BCI research group has proposed a novel speller paradigm called “Hex-o-Spell,” with which subjects spell a character using two-step commands in a hexagon interface (Blankertz et al., 2006). For most subjects, the speed of their performance in this two-class MI-based BCI speller was between 4.6 and 7.6 LPM. However, the performances of existing MI-based spellers are not comparable with visual stimuli-based speller (Blankertz et al., 2006; D'albis et al., 2012). Recently, the efficiency of MI-based BCI speller has been greatly improved by the Oct-o-spell paradigm in our previous work (Chen et al., 2013). We developed a 2-D cursor control strategy using the modified Hex-o-Spell paradigm and an asynchronous control switch was used for starting the system.

In this paper, synchronous protocol is presented for BCI speller. Furthermore, an intelligent input method is used for improving the performance of the BCI speller. The online experiment is conducted involving three subjects. The comparable result is showed to validate the feasibility of this BCI speller.

## 2. Methods

### 2.1. Systematical structure

#### 2.1.1. Oct-o-spell paradigm

**Figure 1:**
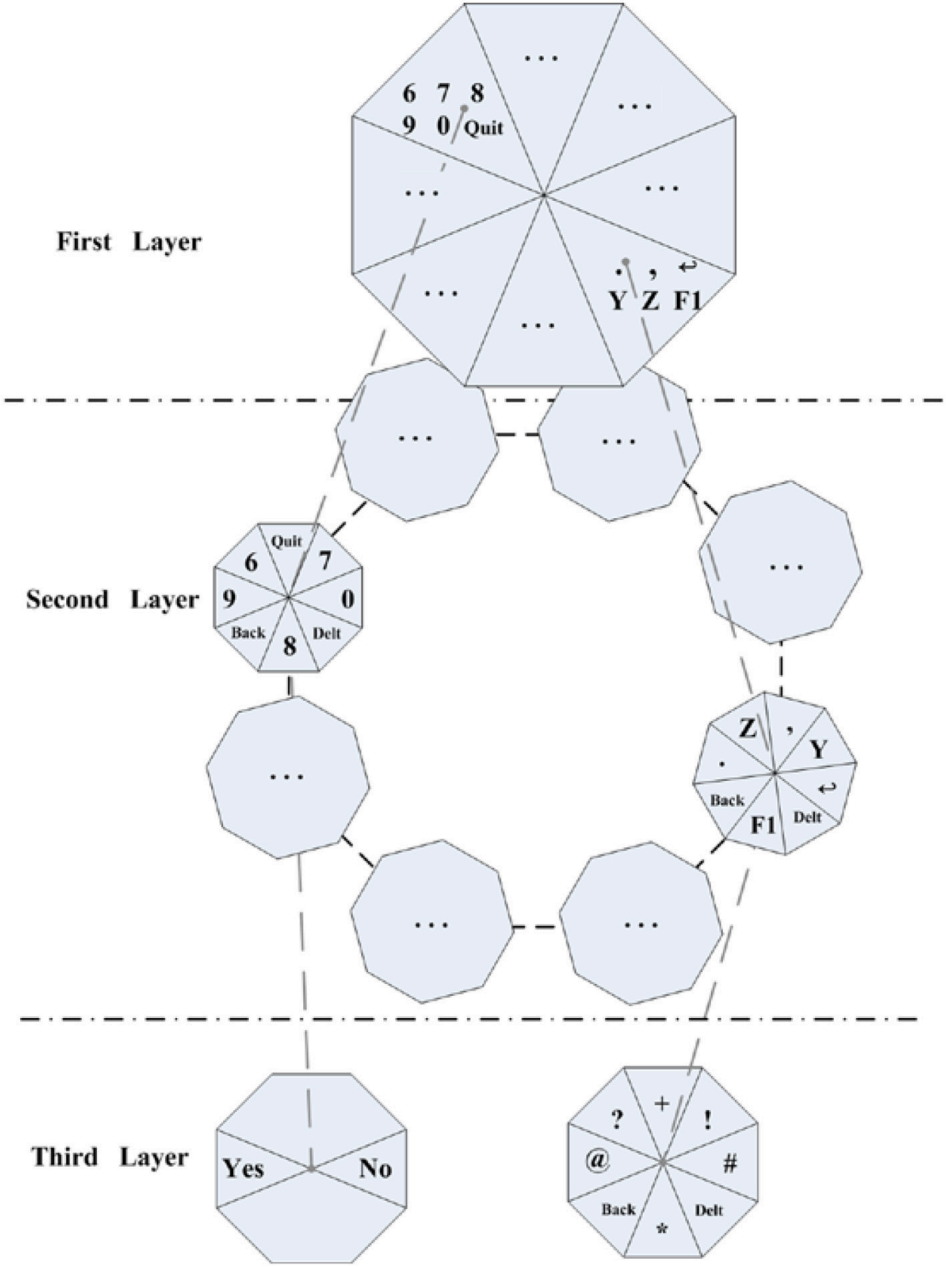
**In the first layer, 26 letters, 10 digits, and 6 symbols are divided into eight blocks**. In the second layer, 7 different interfaces are designed to unfold the blocks in the above layer. Only two symbols are connected to the third layer. The subject can select the associating word by numeric option layer by layer.

In this paper, a three-layer interface of Oct-o-Spell paradigm is proposed for character input (Figure 1). In the first layer, 26 letters, 10 digits and 6 symbols (“Quit,” “=,” “←,” “.,” “F1,” “Chn/Fn”) are divided into eight blocks. In the second layer, 8 different interfaces are designed to unfold the blocks in the first layer. Only two symbols are connected to the third layer. One is “F1,” including 6 punctuation: “^*^,” “@,” “?,” “+,” “!,” and“#.” The other one is 'Quit', which is used for quitting the system. After the user inputs several characters, associative words will be listed by numeric labels. The user can select it by entering the corresponding number. Then, he/she doesn't need to type all letters for saving considerable time. The function is adopted for predictive text entry (PTE) to improve the efficiency of character input. The monitor resolution is 1,440^*^838 pixels and the ratio of the size of cursor target circle and the screen is 0.0003:0.0065:1.

#### 2.1.2. Motor imagery-based synchronous control protocol

**Figure 2:**
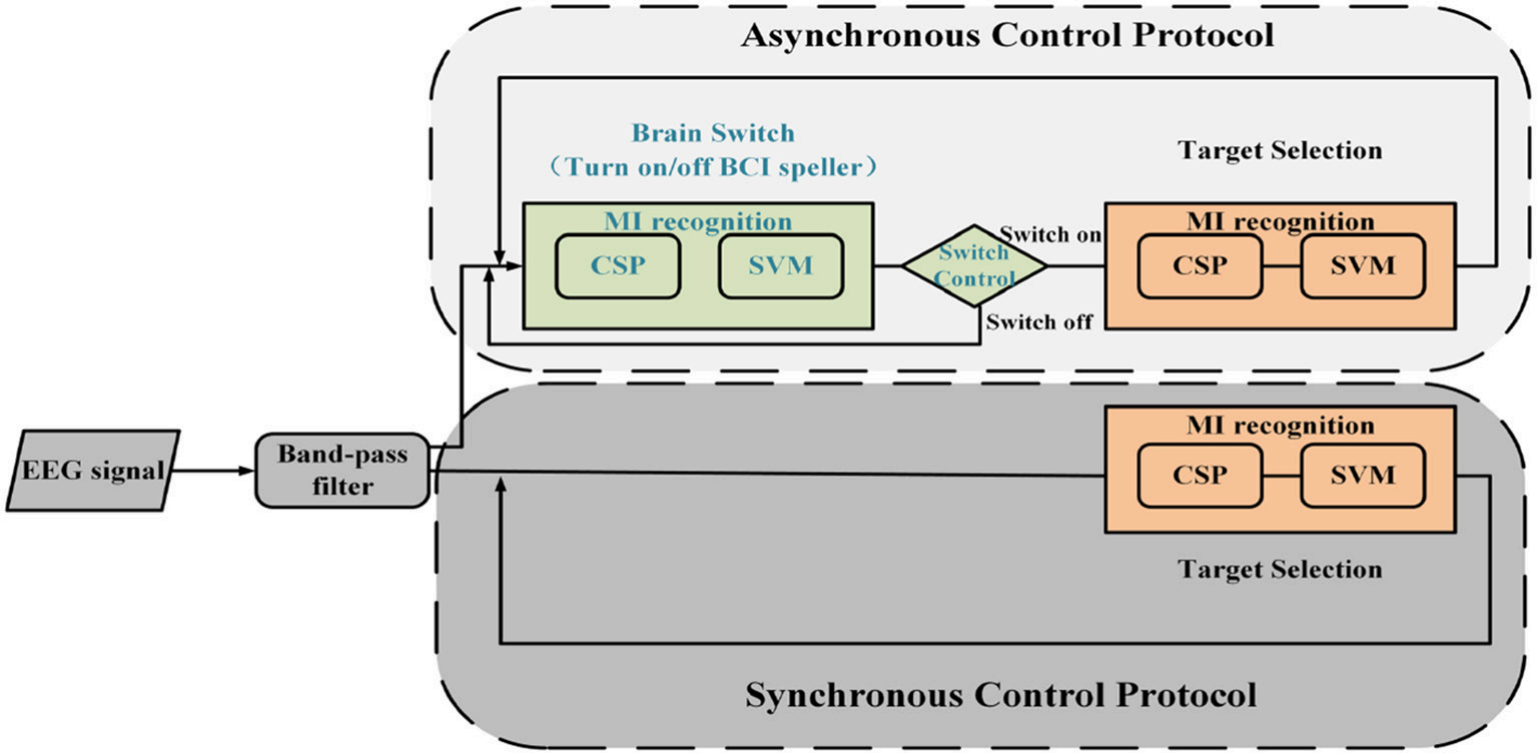
**Framework for asynchronous and synchronous control protocols**. First, EEG signals are band pass filtered at 5–30 Hz for removing background noise. Then, common spatial pattern (CSP) and support vector machine (SVM) are combined to discriminate three-class (left hand, right hand, foot) MI patterns. For asynchronous control protocol, a brain switch is used for turn on/off this BCI speller by MI tasks.

**Figure 3:**
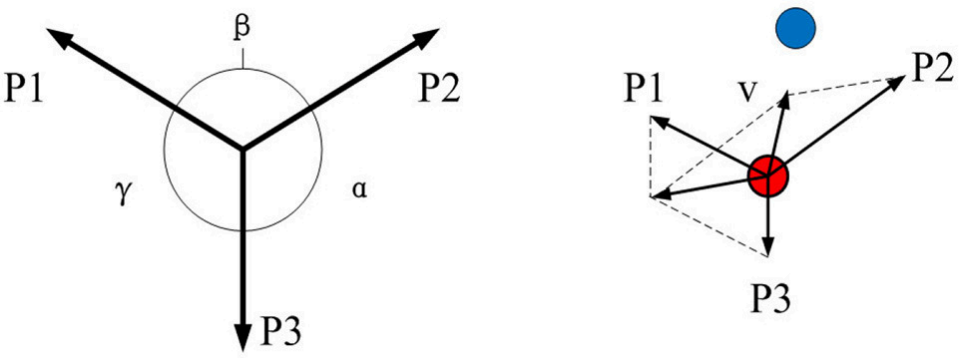
**The diagram of MI-based control vector decomposition**. The classification probabilities are marked as P1, P2, P3 which is mapped to three vectors. The angle between each two vectors is 120° and the length of the vector is equal to the value of the probability.

In the previous study (Chen et al., 2013), we presented a motor imagery-based BCI for asynchronous 2D cursor control. Compared with this method, synchronous control remove the brain-actuated switch to improve the efficiency. These two control protocols are illustrated in Figure 2. First, EEG signals are band pass filtered at 5–30 Hz for removing background noise. Then, common spatial pattern (CSP) and support vector machine (SVM) are combined to discriminate three-class (left hand, right hand, foot) MI patterns. The CSP method is useful for discriminating two populations of EEG. Based on the dataset, a transformation matrix is calculated for a spatial filter. The first and last rows of the CSP transformation matrix could maximize the difference of two groups of data. In our algorithm, the first and last two rows are selected to construct the transformation matrix for further data analysis. On the other hand, the probabilities from SVM classifier are three decimals ranged from 0 to 1 and sum of them equals to 1. The feature vectors are fed into the SVM classifier, provided by LIBSVM toolbox (Chang and Lin, 2011), to predict the class label and probability estimate for multi-class task. The radial basis function (RBF) is selected as the kernel function and a five-fold cross-validation in training data is used for choosing suitable SVM parameters. As shown in Figure 3, they are marked as P1, P2 and P3 which are mapped to three vectors. The angle between each two vectors is 120° and the length of the vector is equal to the value of the probability. To hit the target located between two vectors, the user performs two corresponding motor imagery tasks simultaneously to generate a resultant vector, by which the cursor is moved directly to the target.

On the other hand, the moving distance is computed by the classification result. We define the moving step as L, and the horizontal and vertical moving distances, *d*_*x*_ and *d*_*y*_, are calculated as follows:

(1) $$\begin{array}{r} d_{x}=\left(p2-p1\right)\times cos30{}^{\circ}\times L \end{array}$$

(2) $$\begin{array}{r} d_{y}=\left[\left(p1+p2\right)\times sin30{}^{\circ}-p3\right]\times L \end{array}$$

where the negative value means that the cursor moves left and vice visa.

**Figure 4:**
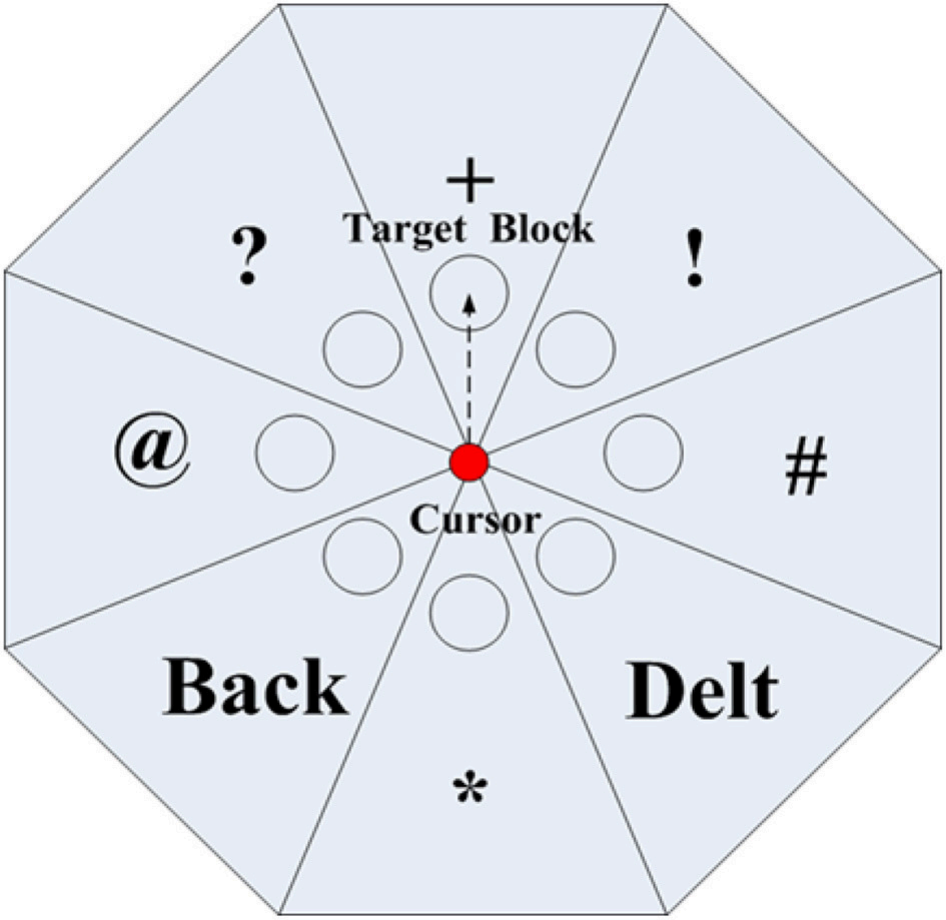
**The user performs MI tasks to drive the cursor to the circle of the target block**. The corresponding character will be selected for output.

To spell character or exit this system, the subject needs to move the cursor to the target circle of the block in each layer. At the first layer, the user can move cursor to reach the target circle of the characters block (containing the target character). Then the interface will extend to the second layer according to the previous selection. Then, the target character can be targeted in the second layer and the speller will output this letter. Only “Quit” or “F1” is chosen in this layer, the interface would extend to the next layer. And the target character can be typed in the third layer or quit the system. To hit a target block (Figure 4), the user should perform MI tasks to drive the cursor to the circle of the target block. If a wrong block is selected, the “Back” command in the next layer can be selected to return to the previous interface. If a wrong character is chose, the subject can select the “Delt” command to delete it in the second layer. The MI BCI system will produce projection values for cursor moving every 0.5 s. Typically, the subject needs to take about 3 s (6 trials) to conduct MI task for targeting the circle of a block in one certain layer. And the maximum time consumption will be given in the description of experimental settings.

### 2.2. Subjects and experimental settings

Six healthy subjects (4 males, 2 females) aged between 19 and 26, took part in our experiment. All of them were naive in BCI and consent to be involved in the study. Firstly, three-class (left hand imagery, right hand imagery and feet imagery) MI sessions were conducted for training in one day. Each training session included 20 trials for each class and each trial last 2 s. Subjects had a short break between sessions. In the experiment, each subject was seated in front of a notebook computer, with arms on the chair arms and hands relaxed. An arrow pointing three different directions was used to indicate one class MI. Finally, three subjects who had achieved performance rates of over 70% (criterion level) were selected for subsequent spelling experiment. In the spelling experiment, all previous train data were used for training SVM classifiers. EEG signals were recorded by a 16-channel g.USBamp amplifier, and 13 electrodes were placed according to the international 10–20 system (FC3, FCZ, FC4, C5, C3, C1, CZ, C2, C4, C6, CP3, CPZ, and CP4). The ground and reference electrodes were placed on the FZ position and the right earlobe. EEG signals were sampled at 256 Hz and band-pass filtered between 5 and 30 Hz.

Online copy spelling experiment: In this experiment, the subject used the above synchronous method for MI-based BCI task. This BCI system could produce three projection values in corresponding directions every 0.5 s for cursor moving. The BCI classifier model was trained by previous MI training sessions. In order to help the subjects with the speller interface, each subject performed 20 free spell familiarize runs in which subject was asked to type characters from A to Z (26 letters) for 2 days. Then the subjects participated in an online copy spelling experiment on the third day. Firstly, 3 subjects spelled 15 English words by PTE and non-PTE modes. The words were selected from reference (Hwang et al., 2012): “WOMEN, DESK, WATER, HAND, MEMORY, ZONE, BABY, FACE, TAXI, JUNE, QUICK, VIDEO, GOLF, HOUR, PENCIL” for synchronous paradigms testing. There was a 3-s interval between trials. If the subject couldn't spell the word correctly in 40 s, this trial would be ended and another trial started. Wrongly typed letters had to be corrected. Furthermore, all subjects performed Chinese character input by PTE mode. Ten common words were used for this test (Table 1). And the rule was the same as English character input.

**Table 1:** **The Chinese words and their translations used for spelling experiment**.

How are you	Thirst	Do you have a meal	Good morning	Sorry
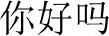	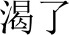	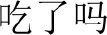	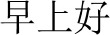	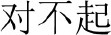
Good night	Never mind	Thank you	Remember	Cannot hear
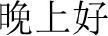	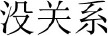	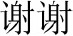	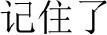	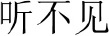

## 3. Results

In our study, online English and Chinese words spelling experiments were respectively conducted to evaluate our synchronous BCI system. Conventionally, some typical indicators were selected to assess the systematical efficiency. (1) Accuracy rate (ACC): the percentage of successful classification result. (2) Information transmission ratio (ITR): the bit rate of information communicated per unit time. (3) Letter per minute (LPM).

Table 2 shows the performance for 3 subjects. The comparison between PTE and non-PTE methods didn't show significant differences.

**Table 2:** **The spelling results between Non-PTE mode and PTE mode for all 3 subjects**.

**Mode**	**Non-PTE**			**PTE**		
**Indicator**	**ACC (%)**	**ITR**	**LPM**	**ACC (%)**	**ITR**	**LPM**
Sub 1	100	90.38	15.05	95.2	56.86	15.62
Sub 2	94.0	53.62	6.33	95.5	59.13	6.77
Sub 3	99.0	63.49	7.6	99.2	71.19	8.07
Mean	98.3	69.16	9.66	96.6	62.39	10.15

**Figure 5:**
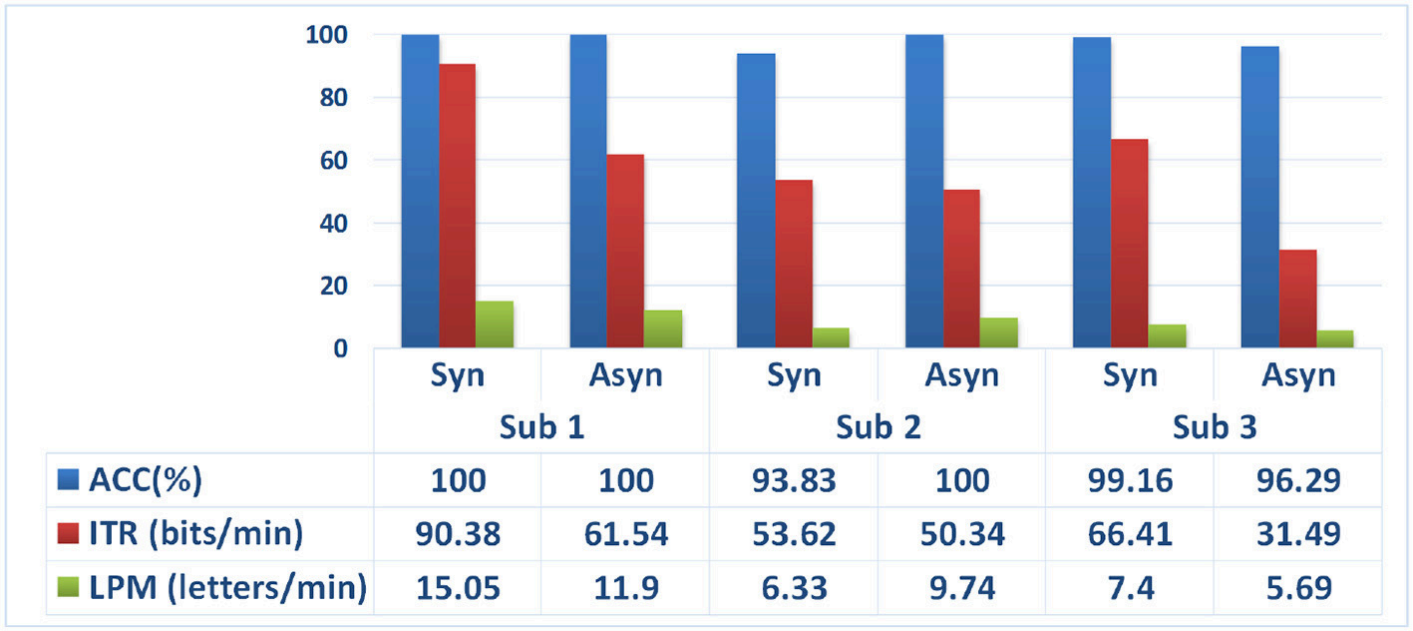
**The accuracies, ITRs and LRMs for 3 subjects by synchronous mode and asynchronous mode**.

For the English word spelling experiment, we compared synchronous control with previous asynchronous control under the same experimental condition (Figure 5). There were no significant differences between these two control methods in ACC (paired *t*-test: *t* = 0.4125, *p* > 0.05), ITR (paired *t*-test: *t* = 2.3053, *p* > 0.05) or LPM (paired *t*-test: *t* = 0.2428, *p* > 0.05). Even so, the synchronous mode had better performance in ITR and LPM. It was verified that this strategy had higher efficiency in BCI output than that of asynchronous mode.

Chinese spelling tasks were performed by all subjects. The performance is presented in Table 3. All subjects achieved sufficient performance rate of over 70% (criterion level). It demonstrated that our proposed BCI speller was feasible for both English and Chinese.

**Table 3:** **The experimental performance of Chinese words spelling for 3 subjects**.

**Indicator**	**Accuracy (%)**	**LPM**	**ITR**
Sub 1	82.82	5.41	34.76
Sub 2	94.94	5.22	48.02
Sub 3	98.65	7.4	66.41
Mean	92.13%	6.01	49.73

## 4. Discussion

For 3 subjects, experimental results showed that motor imagery was feasible for common communication and expression in BCI. All subjects gave positive feedback to the manipulation of the speller after experiments. No pronounced difference was observed between our MI-based speller and other BCI spellers (e.g., SSVEP, P300) (Cecotti, 2010; Jin et al., 2011a,b; Xu et al., 2014). Moreover, the accuracy and ITR of our MI-based speller are approximately equal to those of hybrid BCIs (Xu et al., 2013; Yin et al., 2013). This MI-based control strategy is useful for Oct-o-Spell paradigm. The result of LPM implied that our MI-control strategy had better output performance than those of previous BCI spellers (Blankertz et al., 2006; D'albis et al., 2012). Moreover, the number of candidate characters was 36 for our BCI system. And previous MI-based BCI spellers had few target characters for user selection. It indicated that our BCI speller was more practical than other MI-based BCI spellers.

**Figure 6:**
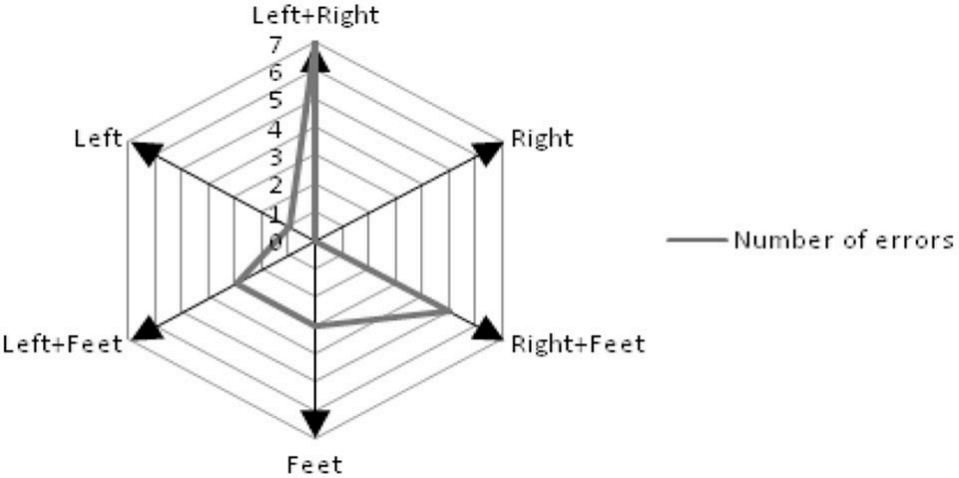
**The number of errors for all kinds of motor imagery including left hand motor imagery (Left), right hand motor imagery (Right), feet motor imagery (Foot), left hand and feet motor imagery (Left + Feet), right hand and feet motor imagery (Right + Feet) and left hand and right hand motor imagery (Left + Right)**.

In the experiment, subjects performed two motor imagery tasks simultaneously. Compared with traditional single modality-based MI control task, it was more difficult to obtain accurate results in this parallel control system because of simultaneous multi modality -based MIs. We reported the number of errors for all motor imagery tasks (Figure 6). The error was defined as the incorrect block selection resulted from cursor moving, which indicated by the use of “Back” and “Delt.” It was speculated that, before these two characters were used for correcting a mistake, the user didn't move the cursor along with the target direction, caused by poor performance in MI tasks. It indicated that synchronous motor imagery tasks (i.e., left hand and feet motor imagery, right hand and feet motor imagery, and left hand and right hand motor imagery) were harder to be mentally controlled. Hand-based motor imagery with two hands was the most difficult task for moving the cursor to the target point. Therefore, characters used frequently must be located in the position where the subject is able to move the cursor by single modality-based MI.

Although the precision and ITR of Non-PTE mode was slightly higher than PTE mode, no significant differences were observed in terms of performance between the PTE and the Non-PTE mode. The detailed process of character input for all subjects was reported at Tables 4–6. Although there were more spelling errors in the PTE mode, which was probably due to distraction caused by word selection, the number of required characters by the PTE mode was less than that for the Non-PTE mode. Nevertheless, this disadvantage was compensated by the reduction of required spelling letters. It was reflected in the average number of spelling letters per word. That was the reason why the LPM of the PTE mode was higher than that for the Non-PTE mode. Moreover, the efficiency of PTE mode could be improved by raising the precision of character selection.

**Table 4:** **The detailed performance of character input for Subject 1**.

**Mode**	**Non-PTE**		**PTE**	
**Word**	**Performance of Subject 1**			
women	women	women	←wo4	←wo4
desk	desk	desk	des4	des4
water	water	water	wa2	r←wa2
hand	hand	hand	ha5←←←ha6	ha(back)6
memory	memory	memory	me2	me2
zone	zone	zone	z3	z3
baby	baby	baby	ba2	ba2
face	face	face	fa4	fa4
taxi	taxi	taxi	r←h←tax3	taxi1
june	june	june	ju4	j(back)9
quick	quick	quick	q3	q3
video	video	video	v1	v1
golf	golf	golf	g7	g7
hour	hour	hour	hou3	hou3
pencil	pencil	pencil	pen6	pen6
Mean accuracy	100%	100%	93.64%	96.72%
LPM(letters/min)	14.53	15.56	15.43	15.79
ITR(bits/min)	87.39	93.36	55.55	58.17
Average number of spelling letters per word	4.53	4.53	3.73	3.13

*‘←’ means · Delt'*.

**Table 5:** **The detailed performance of character input for Subject 2**.

**Mode**	**Non-PTE**		**PTE**	
**Word**	**Performance of Subject 2**			
women	women	women	w(back)o4	3←wo4
desk	des(back)k	desk	(back)des4	desk(back)(back)4
water	water	water	wa2	wa2
hand	(back)hand	hand	ha6	ha←(back)and1
memory	md←emory	memory	me2	me2
zone	←zone	zone	zo2	z3
baby	baby	baby	ba2	ba2
face	face	face	fa4	fac2
taxi	taxj←i	taxi	tax3	tax3
june	(back)june	june	ju4	(back)ju4
quick	quick	quicl←k	q3	q3
video	s←video	video	vbn←←(back)1	v1
golf	gol←←j←olf	golf	go3	go3
hour	hour	(back)hour	hou3	(back)hou3
pencil	pencif←l	pencil	pen6	pen6
Mean accuracy	89.94%	97.71%	96.12%	94.96%
LPM(letters/min)	5.14	7.52	6.35	7.18
ITR(bits/min)	50.46	56.77	69.87	48.37
Average number of spelling letters per word	5.73	4.67	3.4	3.6

*‘←’ means · Delt'*.

**Table 6:** **The detailed performance of character input for Subject 3**.

**Mode**	**Non-PTE**		**PTE**	
**Word**	**Performance of Subject 3**			
women	women	women	wo4	wo4
desk	desk	desk	des4	des4
water	water	water	wa2	wa2
hand	han(back)d	hand	ha6	h(back)a6
memory	memory	memor←ry	m(back)e2	me2
zone	zone	zone	z3	z3
baby	baby	baby	ba2	ba2
face	face	face	fa4	fa4
taxi	taxi	taxi	r←h←tax3	taxi1
june	june	june	ju4	jun2
quick	quick	quick	q3	qu3
video	video	video	v1	v1
golf	golf	golf	g(back)7	g(back)o3
hour	hour	hour	hou3	hou3
pencil	pencil	pencil	pen6	pen6
Mean Accuracy	99.33%	99.05%	96.12%	98.33%
LPM(letters/min)	6.01	9.19	6.55	8.58
ITR(bits/min)	62.39	64.57	72.41	69.96
Average number of spelling letters per word	4.53	4.67	3.13	3.2

*‘←’ means · Delt'*.

The ITRs for the synchronous mode were higher than those for the asynchronous mode for all the subjects. The asynchronous BCI took some time to start the speller, which reduced the transmission efficiency in a trial. Therefore, the number of switch control was decreased for improving the efficiency of the BCI speller. Therefore, subjects needed to reduce the use of switch control for improving the efficiency of the BCI speller. For instance, when the user activated the switch consciously in the first layer, the switch would be active automatically for target selection in the second and third layer. Because the user intended to spell something after activating the speller in the first layer. The switch was redundant to detect mental state when the user was conscious to manipulating. Thus, the time consumption would be reduced for better ITR and LPM. Meanwhile, we could add a selective function for switching off the system in the second and third layers. It was used for correcting the false positive selection in the first layer. This control protocol that possess the superiority of both patterns, would be developed in further work.

The number of electrodes of our BCI speller was higher than SSVEP-based BCI speller (Xu et al., 2014). As mentioned, fewer channels were beneficial for wearable portable BCI devices. And the selection of electrodes would be considered for real conditions and user experience in future. Furthermore, optional mode-based BCI speller should be developed to account for individual differences.

## 5. Conclusions

This paper proposed a novel MI-based BCI speller with Oct-o-spell paradigm for word input. The 3D motor imagery was used for synchronous control. The detailed analysis was given in the discussion. Our results showed that MI-based synchronous protocol was feasible for BCI speller. And the efficiency of the PTE mode was superior to that of the non-PTE mode.

## Ethics statement

This study was carried out in accordance with the recommendations of “name of guidelines, name of committee” with written informed consent from all subjects. All subjects gave written informed consent in accordance with the Declaration of Helsinki. The protocol was approved by the “name of committee.”

## Author contributions

LC and BX contributed to the conception of the study, as well as performed the data analyses and wrote the manuscript. OM, JL, and HX contributed significantly to analysis and manuscript preparation. And NB helped revising the manuscript critically for important intellectual content.

### Conflict of interest statement

The authors declare that the research was conducted in the absence of any commercial or financial relationships that could be construed as a potential conflict of interest.
